# Phase II study of docetaxel in combination with epirubicin and protracted venous infusion 5-fluorouracil (ETF) in patients with recurrent or metastatic breast cancer. A Yorkshire breast cancer research group study

**DOI:** 10.1038/sj.bjc.6601840

**Published:** 2004-05-11

**Authors:** A C Humphreys, J Dent, S Rodwell, S M Crawford, J K Joffe, C Bradley, D Dodwell, T J Perren

**Affiliations:** 1 CRUK Clinical Centre in Leeds, St James's University Hospital, Beckett Street, Leeds, LS9 7TF, UK; 2 Airedale General Hospital, Skipton Road, Steeton, Keighley BD20 6TD, UK; 3 Huddersfield Royal Infirmary, Lindley, Huddersfield HD3 3EA, UK; 4 Bradford Royal Infirmary, Duckworth Lane, Bradford BD9 6RJ, UK; 5 Cookridge Hospital, Hospital Lane, Cookridge, Leeds LS16 6QB, UK

**Keywords:** breast cancer, taxotere, epirubicin, infusional 5-fluorouracil

## Abstract

This study was originally designed as a phase I/II study, with a dose escalation of docetaxel in combination with epirubicin 50 mg m^−2^ and 5-fluorouracil (5-FU) 200 mg m^−2^ day^−1^. However, as dose escalation was not possible, the study is reported as a phase II study of the combination to assess response and toxicity. A total of 51 patients with locally advanced or metastatic breast cancer were treated on this phase II study, with doses of docetaxel 50 mg m^−2^, epirubicin 50 mg m^−2^ and infusional 5-FU 200 mg m^−2^ day^−1^ for 21 days. The main toxicity of this combination was neutropenia with 89% of patients having grade 3 and 4 neutropenia, and 39% of patients experiencing febrile neutropenia. Nonhaematological toxicity was mild. The overall response rate in the assessable patients was 64%, with median progression-free survival of 38 weeks, and median survival of 70 weeks. The ETF regimen was found to be toxic, and it was not possible to escalate the dose of docetaxel above the first dose level. This regimen has therefore not been taken any further, but as a development of this a new study is ongoing, combining 3-weekly epirubicin, weekly docetaxel and capecitabine, days 1–14.

The treatment of metastatic or advanced breast cancer has evolved over the last few years, and in the 1990s this was largely due to the introduction of the taxane family of drugs. Docetaxel (taxotere) is a semisynthetic taxane derived from 10-deacetyl baccatin III, a precursor isolated from the needles of *Taxus baccata*. As with the other taxane compounds, it acts by promoting microtubule assembly and inhibiting microtubule depolymerisation. This blocks cells in M phase and so prevents cell division.

Docetaxel has well-documented activity in advanced breast cancer with a single agent dose of 100 mg m^−2^. The dose-limiting toxicity of docetaxel is grade 3 and 4 neutropenia. The response rate ranges from 19–57% in pretreated patients and 54–67% in those with minimal pretreatment (Clemons *et al*, 1997).

Following the single-agent studies, docetaxel was combined with other active agents. The most attractive combination, initially, was with anthracyclines, as docetaxel is known to have activity in anthracycline-resistant disease (Miller *et al*, 2001). Studies were carried out using epirubicin and docetaxel. For example, docetaxel was escalated with a fixed dose of epirubicin (Venturini *et al*, 2001). It was found that the dose-limiting toxicity was neutropenia, which was ameliorated to some extent by G-CSF. Other side effects were mild. There were however two toxic deaths. The recommended doses for further investigation were epirubicin 75 mg m^−2^ and docetaxel 80 mg m^−2^. In a further study (Viens *et al*, 2001), escalating doses of epirubicin were given with a fixed dose of docetaxel 75 mg m^−2^. Dose-limiting toxicities were grade 3 and 4 asthenia, febrile neutropenia and stomatitis and diarrhoea. There was an overall response rate of 69.4%, and a median duration of response of 7.8 months. The recommended dose was epirubicin 100 mg m^−2^ with docetaxel 75 mg m^−2^. An overview of these studies in 2001 (Trudeau and Pagani, 2001) concluded that epirubicin and docetaxel was effective, with high response rates, and no significant cardiotoxicity.

Docetaxel has also been combined with 5-fluorouracil (5-FU). A phase I study (Lortholary *et al*, 2000) escalated doses of docetaxel, given on day 1, along with escalating doses of 5-FU given as a 5-day intravenous infusion, every 3 weeks. The most common toxicity was grade 4 neutropenia, which occurred in 91% of patients. The recommended dose for phase II studies was 85 mg m^−2^ docetaxel with 750 mg m^−2^ day^−1^ 5-FU for 5 days.

At the time this study was initiated, a very active regimen that was used was the ECF regimen of epirubicin, 50 mg m^−2^, cisplatin, 60 mg m^−2^ and infusional 5-FU, 200 mg m^−2^ day^−1^. A phase II study of eight courses of ECF in 43 patients with metastatic and locally advanced breast cancer (Jones *et al*, 1994) gave an overall response rate of 84%. This regimen has also been used in a phase II study in patients with large but operable breast cancer in the neoadjuvant setting (Smith *et al*, 1995). A total of 50 patients were treated with an overall response rate of 98% and 66% complete response rate. Grade 3 and 4 toxicities were rare. The ECF regimen was of sufficient interest to investigate it further in phase III studies in both the neoadjuvant (TOPIC Trial) and high-risk adjuvant (TRAFIC) settings.

The ECF regimen, in general, is well tolerated. However, the inclusion of cisplatin in the regimen usually necessitates an overnight stay for each course of treatment. We therefore looked at the possibility of using epirubicin and 5-FU in the doses used in the ECF regimen, but combining them with docetaxel, a very active agent in metastatic breast cancer, which can be given as an outpatient.

## STUDY OBJECTIVES

The original intention of this study was to perform a dose escalation study of docetaxel in combination with epirubicin (50 mg m^−2^) and continuous infusional 5-fluorouracil (200 mg m^−2^ day^−1^), and then to carry out a phase II study at the defined doses in patients with metastatic or advanced primary breast cancer. However, dose escalation of docetaxel was not possible, due to toxicity, and it is therefore reported as a phase II study.

## PATIENTS AND METHODS

### Study design

This phase II study of the ETF combination was carried out at the initial dose of docetaxel in the phase I design, 50 mg m^−2^, in patients with metastatic or advanced primary breast cancer, to assess response rate and toxicity.

The study was approved by the local ethics committee of each hospital carrying out the study.

### Data collection and statistical analysis

The study was carried out in five different cancer units within Yorkshire. The data was collected and analysed at the cancer centre. Survival and progression-free survival were analysed by the Kaplan–Meier method.

### Patient selection

Eligible patients were 70 years or less with histologically confirmed advanced or metastatic breast cancer. Previous adjuvant or neoadjuvant chemotherapy was permitted, which could be anthracycline based, if completed at least 18 months prior to study entry. Previous endocrine and radiotherapy were permitted.

Patients were to be of Eastern Cooperative Oncology Group (ECOG) performance status 0, 1 or 2 and have a life expectancy of at least 3 months. They must not have uncontrolled brain metastases, have no other serious medical conditions, no history of significant cardiac disease, adequate baseline bone marrow (neutrophils ⩾1.5 × 10^9^ l^−1^ and platelets ⩾100 × 10^9^ l^−1^) and hepatic (normal bilirubin, transaminase ⩽3 times upper limit of normal) function, no previous exposure to taxanes and fully recovered from previous therapies. Patients were not pregnant or lactating.

Most patients had either measurable or evaluable disease. However, those who were entered as part of the phase I study did not necessarily have either measurable or evaluable disease.

All patients were required to give written informed consent prior to entry into the study.

### Treatment plan

All patients required a central line to be inserted prior to chemotherapy to deliver the continuous infusional 5-FU. To reduce the risk of thrombosis, all patients were started on a minimum of warfarin 1 mg day^−1^, but exact warfarin regimens were left to individual units practice.

Docetaxel was given as a 1 h infusion with standard steroid prophylaxis, followed by epirubicin as an intravenous bolus, on day 1. 5-Fluorouracil was given by continuous intravenous infusion via a central line and a portable pump.

In order to proceed to the next cycle of chemotherapy, the required neutrophil count was ⩾1.5 × 10^9^ l^−1^. No G-CSF was used in this study.

### Patient evaluation

Prior to beginning the treatment, all patients underwent a physical examination, including height and weight, a full blood count (FBC), urea and electrolytes (U&E), liver function tests and bone biochemistry. While on study, all patients had weekly FBC. Urea and electrolytes, liver function tests and bone biochemistry were carried out prior to each course of chemotherapy.

Patients underwent a baseline chest X-ray, a bone scan for known or suspected metastases and a CT or ultrasound of the liver. Patients had documentation of other disease sites, either clinically, photographed, or by means of suitable imaging.

Disease response was documented after three and six cycles of chemotherapy.

### Response criteria and end points

The end points for the study were toxicity, response and response duration, where patients had clinically evaluable disease and survival.

Response was assessed after three and six courses of chemotherapy, either clinically or by radiological means. Response was graded by WHO criteria, as complete response (CR), partial response (PR), stable disease (SD) or progressive disease (PD).

### Dose modifications

Toxicity was graded according to the Common Toxicity Criteria.

Haematological toxicity was recorded weekly. Dose-limiting toxicity was defined as grade 4 neutropenia or thrombocytopenia, lasting for 1 week or longer, or with fever (single oral temperature >38.5°C or three temperatures of >38°C within 24 h) lasting more than 2 days. This resulted in a reduction of all drugs by 25% in the next and subsequent cycles. If there was further symptomatic grade 4 haematological toxicity there was a further dose reduction, to 50% of starting dose.

For all grade 3 nonhaematological toxicities, the dose of specific drugs was reduced by 25%. For example, for mucositis, palmar-plantar syndrome or diarrhoea, 5-Fluorouracil dose was reduced by 25%. If grade 3 mucositis occurred despite this dose reduction, then both taxotere and epirubicin were reduced, also by 25%, on the next course of treatment. However, for grade 2 neuropathy, a 25% dose reduction of taxotere was made, and for grade 3 or 4 neuropathy the patient was withdrawn from the study.

Hypersensitivity reactions were treated according to local policy.

## RESULTS

### Patient characteristics

In total, 51 patients were entered onto the study and treated between January 1997 and June 1999.

The median age of the patients was 47 years (range, 33–67). A total of 44 patients (86%) were treated for metastatic disease and seven (14%) had locally advanced breast cancer. As the study was originally set up as a phase I leading to a phase II study, six patients received ETF with nonassessable disease, for example, bone metastases alone, or resected lymph nodes. Eighteen patients (35%) had had no previous treatment for breast cancer in either the adjuvant or metastatic setting; 23 (45%) had received adjuvant chemotherapy (12 anthracycline based and 11 CMF); 25 (49%) adjuvant radiotherapy and 27 (53%) adjuvant endocrine therapy. Of the 51 patients treated, 21 (41%) had liver metastases, 11 (22%) had bone metastases, nine (18%) had locally advanced breast primary, nine (18%) had metastatic lymph nodes and six (12%) had lung metastases.

### Toxicity

In total, 261 courses of ETF chemotherapy were given. The number of patients who received six full courses of chemotherapy, as planned, with no delays was 13 (25%). All other patients required dose reductions, dose delays or the chemotherapy was stopped early because of neutropenic sepsis (11 patients), mucositis (eight), line problems (six), personal reasons (three), progressive disease (two) and others (six), including Herpes zoster infection and a chest infection.

In all, 15% of planned doses were not given, due to toxicity or PD.

### Haematological toxicity

In a small number of cases (18 courses), a weekly full blood count was not carried out.

This chemotherapy regimen was found to cause significant haematological toxicity as 89% patients had grade 3 or 4 neutropenia, with 39% patients experiencing episodes of neutropenic sepsis, Table 1

**Table 1:** Haematological toxicity by patient and by course

Number of patients (%), grade 3 neutropenia	12 (24%)
Number of patients (%), grade 4 neutropenia	33 (65%)
Number of courses (%), grade 3 neutropenia	73 (28%)
Number of courses (%), grade 4 neutropenia	61 (23%)
Number of patients (%), febrile neutropenia	20 (39%)
Number of courses (%), febrile neutropenia	24 (9%)

. In total, 24 of 261 courses of chemotherapy given (9%) were complicated by febrile neutropenia. One patient had grade 4 neutropenia following her first course of ETF, with a white cell count of 0.3 and a neutrophil count of 0.03. She was admitted to hospital and treated with intravenous antibiotics, but was found to have typhilitis, neutropenic enterocolitis, and ultimately died from this. This is an uncommon but recognised complication of patients receiving aggressive chemotherapy, and has been described in patients who have had docetaxel (Cardenal *et al*, 1996). A further patient was admitted to another hospital following her third course of ETF chemotherapy. She was neutropenic and was septic. Despite intravenous antibiotics she became more unwell and subsequently died from the infection.

Only one patient had grade 3 anaemia, and no patient had significant thrombocytopenia.

### Nonhaematological toxicity

The nonhaematological toxicity was, in general, mild, compared to the haematological toxicity and was mostly grade 1 and 2. The most prevalent problems were grades 1 and 2 mucositis (41%), grades 1 and 2 lethargy (29%) and grades 1 and 2 diarrhoea (25%). However, central lines were a problem, being the source of toxicity in 24 (46%) patients in total. The most prevalent problem was line infections, which occurred in 12 patients, and seven lines were removed because of infection. Four patients experienced thrombosis associated with their lines and a further four experienced pain. Two patients had episodes of mediastinitis, which required removal of the line, and one of these received bolus 5-FU for the remainder of the courses.

In relation to the use of docetaxel, two patients developed a photosensitive skin rash, both grade 2, and in one patient the chemotherapy was stopped. One patient developed grade 1 neurotoxicity.

### Response

The median follow-up of all patients from study entry to the date last seen or the date of death was 70 weeks (range 1–242).

Of the 51 patients, nine did not have assessable disease: four patients had bone metastases only, two patients had metastatic disease in lymph nodes that were removed prior to chemotherapy, one patient had one course of ETF and was then changed to FEC because of a central line infection, and two patients died of neutropenic sepsis. There were therefore 42 assessable patients, of whom 27 had a response. Of these, six had a CR and 21 had a PR. This gave an overall response rate of 64% (95% confidence interval, 47–77%). A further nine patients had stable disease and seven (14%) had PD.

The median progression-free survival was 38 weeks, and the median survival was 70 weeks. Of the 51 patients treated, seven have not yet relapsed, six have relapsed but have not died and two patients died of chemotherapy toxicity.

## DISCUSSION

There have now been several studies of epirubicin and docetaxel carried out, but this is the first that we are aware of that combines epirubicin, docetaxel and continuous infusional 5-FU. The intention was to develop a regimen that could be given and managed as an outpatient, but maintained or improved the high response rate seen in the ECF regimen, by using docetaxel instead of cisplatin.

In the dose escalation part of the study, the doses chosen for the first dose level were docetaxel 50 mg m^−2^ combined with epirubicin 50 mg m^−2^ and infusional 5-FU 200 mg m^−2^ day^−1^. In previous studies of epirubicin and docetaxel alone, doses of epirubicin between 60 and 100 mg m^−2^ were successfully combined with docetaxel 75–80 mg m^−2^. In this study, the dose of epirubicin that was chosen was the same as in the ECF regimen and we therefore expected to be able to escalate the dose of docetaxel beyond the first dose level. However, the addition of continuous infusional 5-FU to epirubicin and docetaxel made a significant difference to the dose of docetaxel that was tolerable and dose escalation was not achieved.

This study was therefore a phase II study of the ETF regimen. It was found to be active with 64% of patients having a CR or PR. This is a lower response rate than that found with ECF (Jones *et al*, 1994). However, the confidence intervals for the ETF study were wide (47–77%), and therefore it cannot be directly compared to the ECF data, and the number of patients in both studies was small. The progression-free and overall survival, however, in our study do compare favourably with the ECF study, suggesting that the response and efficacy to ETF is similar to ECF.

The groups of patients treated in the two studies were different. Firstly, the ratio of metastatic to locally advanced disease in the ECF study was 2 : 1, whereas in the ETF study it was 6 : 1, although none of these patients had received prior chemotherapy for metastatic disease, and only 45% had received chemotherapy in the adjuvant setting. In the ECF study, only 20% of patients had received previous chemotherapy, but the authors did admit that their group was highly selected, being relatively young and of good performance status.

Secondly, more patients in the ETF study, 41%, had liver metastases, conferring a worse prognosis, than in the ECF study (21% of all patients treated).

Thirdly, the difference in response rate may also reflect the fact that the dose of docetaxel was unable to be escalated beyond the first dose level, due to the toxicity of the combination. In total, 89% of patients had grade 3/4 neutropenia, 65% grade 4, with 39% patients experiencing at least one episode of febrile neutropenia. Overall, 9% of courses were complicated by febrile neutropenia, and two patients died from toxicity of the chemotherapy.

Central lines were a major source of toxicity in this study, with nearly half of the patients having problems with their lines, probably due to the depth of neutropenia experienced by these patients. The most common problem was infection, but four patients had thrombosis of the lines, despite prophylactic warfarin, and six patients had physical symptoms attributable to the lines.

Given the toxicity that was experienced with both the central lines and with severe neutropenia with ETF, it was felt that this regimen was too toxic to take into the adjuvant setting. A further study in the advanced setting was therefore proposed, and is currently recruiting patients. This involves the use of weekly docetaxel, which is found to have a more favourable toxicity profile in terms of haematological toxicity, 3-weekly epirubicin and oral capecitabine, an oral fluoropyrimidine that is absorbed by the intestinal mucosa and converted to 5-FU in the tissues.

A recent study of 3-weekly docetaxel±capecitabine in 511 patients with metastatic or locally advanced breast cancer showed a significantly improved survival for the combination of drugs. We are now testing whether the addition of epirubicin to docetaxel and capecitabine is feasible, and if so whether it can improve the response rates and survival of patients with metastatic breast cancer.
